# A rapid progression of new metastatic lesion after cytoreductive nephrectomy: A case report

**DOI:** 10.1016/j.eucr.2021.101848

**Published:** 2021-09-10

**Authors:** Steven Steven, Ferry Safriadi

**Affiliations:** Urology Department, Hasan Sadikin Academic Medical Center, Universitas Padjadjaran, Bandung, Indonesia

**Keywords:** Papillary renal cell carcinoma, Cytoreductive nephrectomy, Pulmonary metastasis

## Abstract

A 55-years-old man presented with the chief complaint of right flank mass and hematuria. CT scan revealed clinical T4 RCC. Cytoreductive nephrectomy (CN) was performed with histopathology result of Papillary renal cell carcinoma. A month later, the patient was admitted to the emergency room with severe dyspnea suggesting intrapulmonary metastases. Here we present an unusual case with a new pulmonary metastasis lesion rapidly appear a month after cytoreductive nephrectomy.

## 1 Introduction

Cytoreductive nephrectomy (CN) has been indicated as part of an integrated management strategy in metastatic renal cell carcinoma. Nephrectomy had been performed in mRCC patients largely as a palliative option for pain control, hematuria, and symptoms related to compression of adjacent viscera. For most patient with metastatic disease, cytoreductive nephrectomy (CN) followed by targeted therapy are necessary of palliative purpose. A study by Mickisch et al. showed that time to progression (5 vs 3 months) with a median duration of survival (17 vs 7 months) were significantly better in study patients with cytoreductive nephrectomy than with targeted therapy alone.^1^ Systemic therapy for metastatic RCC is often not accessible in developing countries due to high cost and it was not covered by the national healthcare system. In such limited circumstances, CN became the primary treatment modality even for metastatic RCC.

It was also reported that nephrectomy performed in this measure can result in spontaneous regression of metastasis in 4% cases.^2^ This interesting phenomenon most frequently occurs after nephrectomy in the elderly with pulmonary metastasis, but it was different from our case. Here we present a new pulmonary metastatic lesion renal cell carcinoma that rapidly appears a month after cytoreductive nephrectomy.

## 2 Case presentation

A 55-years-old male presented with right flank mass in the last 5 months. The complaint was accompanied by gross hematuria and intermittent abdominal pain since 4 months ago. On presentation, no abnormality was found during the thoracic examination. Tender right palpable mass was recognized.

**Fig. 1:**
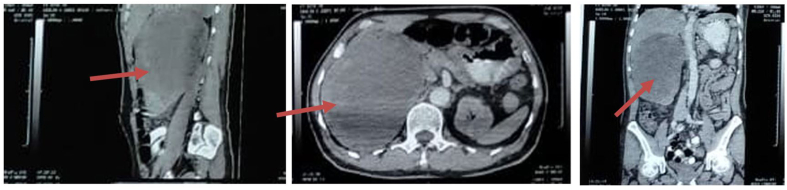
CT Scan of the Abdomen and Pelvic Showed Renal Mass (red arrow). (For interpretation of the references to colour in this figure legend, the reader is referred to the Web version of this article.)

**Fig. 2:**
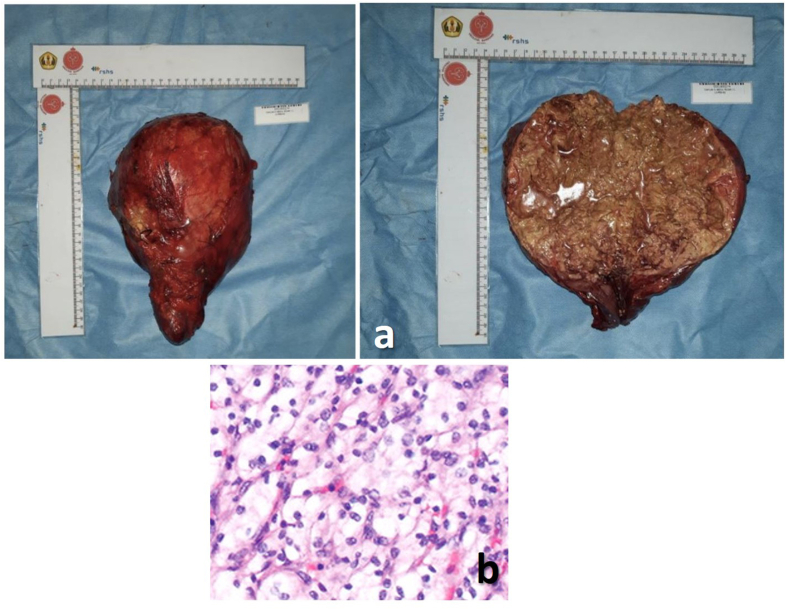
(a)Gross appearance of the RCC, (b) histopathological examination show papillary RCC.

CT scan showed a hypodense lesion that infiltrate the upper pole of the right kidney suggestive clinical T4 renal cell carcinoma without distance metastasis (Fig. 1). We performed explorative laparotomy. Intraoperatively, we found the renal mass was 4 times of normal size, adhere to adjacent organs, and also infiltrate the liver (Fig. 2). We decided to perform cytoreductive surgery with 5 L of blood loss. Histopathology examination suggests papillary cell carcinoma. The patient was discharged after 7 days post-operative and there was no abnormality found during his next outpatient visit.

**Fig. 3:**
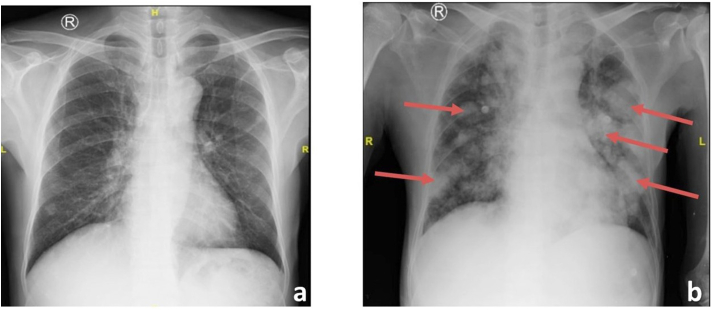
Rontgen Thorax (a) pre-operative, (b) 35 days post cytoreductive nephrectomy showed new coin lesion intrapulmonary (red arrow). (For interpretation of the references to colour in this figure legend, the reader is referred to the Web version of this article.)

Thirty-five days postoperative, the patient presented to the emergency department with severe dyspnea and a decrease of consciousness. Physical examination revealed ronchi in all of pulmonary field, abdomen revealed post-op wound at regio midline abdominal, without any leakage and tenderness. Chest x-ray showed multiple nodular opacity features of various sizes in the upper lung field to the lower lung bilaterally, suggesting rapid progression of intrapulmonary metastases as shown in Fig. 3 with a decrease of consciousness suspected due to brain paraneoplastic syndrome with a history of cytoreductive nephrectomy 35 days post-operation. The patient died thereafter due to severe hypoxia and respiratory failure.

## 3 Discussion

RCCs account for 90–95% of malignant neoplasms arising from the kidney. Notable features include resistance to cytotoxic agents, infrequent responses to biologic response modifiers such as interleukin (IL)-2, and a variable clinical course for patients with metastatic disease, including anecdotal reports of spontaneous regression.^3^ A median time for progression to metastasis after a patient undergo nephrectomy for clinically localized RCC was 1.3 years.^1^

Spontaneous regression of distant metastatic disease after debulking nephrectomy is a phenomenon that has been described in the literature since 1928 by Bumpus. Spontaneous regression of metastatic disease after surgery or after discontinuation of medical therapy suggests that the immune system plays a role in this rare occurrence.^3^ Historically, the principle behind cytoreductive nephrectomy as a treatment for metastatic RCC was based on the immunologic phenomenon of ‘‘spontaneous’’ regression of metastasis after nephrectomy. However, in a review of 474 patients with metastatic disease who underwent nephrectomy alone, only 4 (0.8%) experienced spontaneous regression of their metastatic disease.^3^ Widely accepted historical indications for nephrectomy for metastatic RCC have been to improve quality of life. Surgery may also be directed at metastases to control local symptoms, which include the relief of spinal cord compression and fixation of fractures. Although nephrectomy alone for metastatic RCC was widely discredited, with the emergence of modern immunotherapy in the 1980s and 1990s, the role of nephrectomy and the relative efficacy of initial biological response modifier treatment versus nephrectomy reemerged as a source of controversy.^2^

Rapid progression showed, in this case most likely happened due to several factors. Firstly, in hypoxia condition, the von Hippel-Lindau gene is mutated and Hypoxia-inducible factors (HIF)-α is accumulated in the cytoplasm as well as in the nucleus. The HIS subunit *trans*-activates the target gene especially EPO which form EPOR. This protein participates in tumor progression by upregulating the production of VEGF, increase inflammatory reaction, and decrease intrinsic drug-induced apoptosis and in the end, increase tumor progressions and metastases. Another possible reason is due to tumor resection. Unavoidable damage to tissue during excision and tumor manipulation along with its vasculature can induce shedding of tumor cells into blood and lymphatic circulation. Surgery may also prompt immune escape by triggering postoperative downregulation of adaptive immune such as dendritic cells which decrease tumor removal.^3^^,^^4^

Careful patient selection for CN is critical because those with poor survival outcomes or who are likely to progress rapidly may receive minimal benefit. At least 4 identified preoperative factors such as Karnofsky performance status, hemoglobin, neutrophil, and Clinical N stage used for the selection of patients and those with 0 or 1 risk factor can derive benefit. Patients with estimated survival time <12 months, 4 or more IMDC, more than 3 site-specific metastases, or those with minimal cT3 stage may not receive a substantial benefit from CN. It is very important to select a more appropriate patient to receive CN.^4^^,^^5^

## 4 Conclusion

In patients with RCC, the CN is one of the important parts of treatment. However, in the modern therapeutic era, due to the discovery of targeted agents, an argument has been started on whether the CN is necessary in patients with metastatic RCC recently. Nephrectomy is an independent predictor of overall survival both in conjunction with subsequent therapy or if a watch and wait approach is taken. Nephrectomy was shown to improve survival in wisely selected patients.
